# Supplementary material for: MIK2 is a candidate gene of the S-locus for sporophytic self-incompatibility in chicory (Cichorium intybus, Asteraceae)
Source: Front Plant Sci. 2023 Jun 2;14:1204538. doi: 10.3389/fpls.2023.1204538 (PMC10272723; doi:10.3389/fpls.2023.1204538)
Supplement: Supplementary file 1 [file DataSheet_1.pdf]

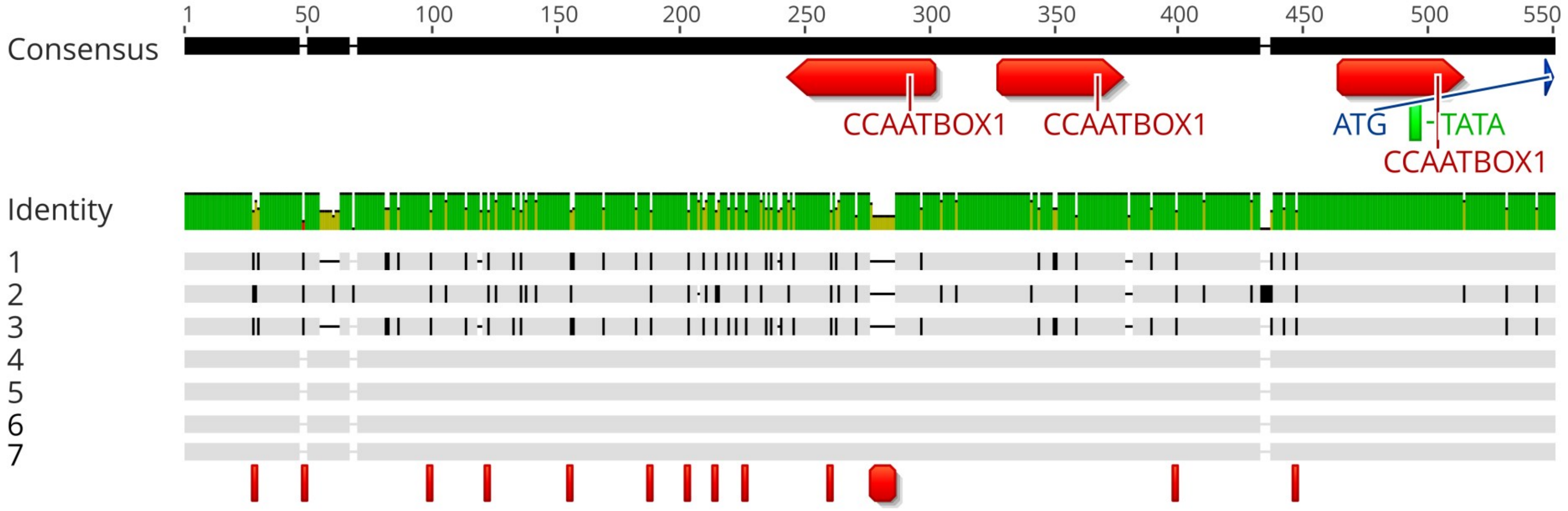

Supplementary Figure 1. Multiple alignment among the first ~500 bp of the MIK2 promoter sequences obtained from 3 chicory and 3 endive accessions. Sample 1-3 are all Radicchio biotypes belonging to *C. intybus* var. *foliosum* (1 and 2 Variegato di Castelfranco, 3 Treviso Precoce). Samples 4-5 are accessions from *C. endivia* var. *latifolium*, sample 6 belongs to *C. endivia* var. *crispum*. Along with the 6 newly obtained sequences, we also included the promoter sequences of MIK2 extracted from the endive genome (Sample 7, *C. endivia* var. *crispum* Fan et al., 2022). All the variants (SNPs and INDELs) detected among the seven sequences are highlighted as black blocks, whilst red blocks indicate species-specific variants (i.e. conserved within species but variable between chicory and endive).
